# Analyzing the Associations Between Mediterranean Diet Adherence, Body Mass Index, and Physical Performance in Youth Handball Players: A Clustering Approach

**DOI:** 10.3390/sports14020075

**Published:** 2026-02-07

**Authors:** Silvia Sánchez-Díaz, Daniel Castillo, Miguel Ramirez-Jimenez, José María Izquierdo, Diego Marqués-Jiménez, Pedro Duarte-Mendes, Marta Domínguez-Díez

**Affiliations:** 1 Departamento de Educación y Formación del Profesorado, Facultad de Ciencias Jurídicas, Educación y Humanidades, Universidad Europea de Madrid, 28670 Villaviciosa de Odón, Spain; silvia.sanchez@universidadeuropea.es; 2 Valoración del Rendimiento Deportivo, Actividad Física y Salud y Lesiones Deportivas (REDAFLED), Department of Didactics of Musical, Plastic and Corporal Expression, Faculty of Education, University of Valladolid, 42004 Soria, Spain; miguel.ramirez.jimenez@uva.es (M.R.-J.); josemaria.izquierdo@uva.es (J.M.I.); diego.marques@uva.es (D.M.-J.); 3 Department of Sports and Well-Being, Polytechnic University of Castelo Branco, 6000-084 Castelo Branco, Portugal; pedromendes@ipcb.pt; 4 Sport Physical Activity and Health Research & Innovation Center (SPRINT), 6000-084 Castelo Branco, Portugal; 5 Department of Didactics of Musical, Plastic and Corporal Expression, Faculty of Education, University of Valladolid, 42004 Soria, Spain; marta.dominguez.diez@uva.es

**Keywords:** handball, youth, nutrition, neuromuscular, clusters

## Abstract

Background: Nutrition is a fundamental factor in the healthy growth and development of young athletes, as well as in supporting optimal sports performance. This study aimed to explore associations between Mediterranean diet adherence score, BMI and selected physical performance measures in youth handball players, by identifying distinct player profiles through a clustering approach. Methods: Thirty-five male youth handball players participated in the study. Mediterranean diet adherence was evaluated by means of a 16-item KIDMED questionnaire and total score, and physical performance was assessed using the countermovement jump (CMJ) test, the 505-change of direction test, linear straight sprints and isometric handgrip strength. Results: Cluster 1 goes more than one day a week to a fast-food restaurant, skips breakfast on more occasions and consumes sweets and candy more often. In addition, Cluster 2 showed better sprint (*p* = 0.019–0.053, ES = 0.39–0.47) and CMJ (*p* = 0.042; ES = 0.40) performance than Cluster 1. Conclusions: These findings present associations between dietary adherence, BMI, and selected physical performance measures in this specific cohort. Given the cross-sectional design and the small sample size, these findings should be interpreted with caution and do not allow causal inferences.

## 1. Introduction

Nowadays, obesity in childhood and adolescence has become a major public health concern worldwide [1]. The growing availability and consumption of energy-dense, nutrient-poor foods, commonly referred to as “fast food”, have significantly contributed to the worsening of this issue [2]. These factors are linked to a higher likelihood of developing conditions such as hypertension, type 2 diabetes, and, in the long term, heart disease and certain types of cancer [2]. Among various healthy dietary patterns, the Mediterranean diet has gained recognition for its health benefits [3]. This eating pattern is characterized by using olive oil as the main fat source, a high intake of seasonal fruits and vegetables, legumes, lean meats and fish, whole grains, and nuts as the foundation of the diet; and by reducing the regular consumption of red and processed meats, ultra-processed foods, and sweets [1,3]. Following this dietary pattern reduces the risk of noncommunicable diseases such as metabolic syndrome, hypertension, and cardiovascular diseases [4,5]. Likewise, it could be relevant for athletes to maintain good nutritional habits, as this could help improve physical performance, because an adequate diet provides the necessary energy to compete, and a lack of calories, carbohydrates, fluids, proteins, vitamins, and minerals can lead to fatigue and poorer physical performance [6].

The body mass index (BMI) is a popular and reliable anthropometric indicator that is easy to calculate, as it only requires height and weight, and is used to assess the nutritional and health status [7]. In this sense, previous studies have found that adolescents with worse nutritional habits could have a higher BMI, which could imply negative health issues [8,9]. In addition, other authors reported that healthy nutritional patterns were correlated with a lower risk of metabolic syndrome and with adequate BMI [10]. In this way, poor nutritional habits may lead to increased BMI and unfavourable cardiovascular profiles [11]. Therefore, the Mediterranean diet could be a key strategy to get reductions in BMI compared to other diets [12]. In sports contexts, literature has shown that adherence to the Mediterranean diet is associated with improved muscular power and endurance, favourable body composition in athletes, and positive impacts on cardiovascular health, cognitive function, and general well-being [13,14]. Mechanistically, the MD provides antioxidants, anti-inflammatory nutrients, and high-quality proteins that support muscle function and neuromuscular adaptations [15]. Evidence also suggests that higher MD adherence is associated with better functional performance, such as walking speed and knee muscle strength, in older adults [16]. Nevertheless, it would be interesting to investigate the impact of the Mediterranean diet on physical performance in various sports modalities, such as handball, and in youth athletes, because of its importance for higher standards of play in their sporting careers.

Handball is a high-intensity, intermittent sport requiring repeated accelerations, jumps, and changes of direction, where neuromuscular performance—speed, power, and agility—is essential for success [17]. These performance components are interrelated and strongly influenced by body composition: greater lean mass supports force production, while excess fat mass impairs relative power and movement efficiency [18]. Thus, dietary habits is essential to help youth athletes enhance their physical performance and perform optimally in their sporting activities. Maughan & Shirreffs [19] reviewed several nutritional strategies that athletes at all levels can implement to optimize performance, highlighting the importance of tailored dietary plans. In handball, physical performance depends heavily on the ability to accelerate, decelerate, change direction, jump, and generate both lower and upper body power, all of which are influenced by an athlete’s nutritional status [20]. Key strategies include ensuring adequate carbohydrate intake to sustain energy during prolonged high-intensity efforts, such as covering long distances in a match, consuming sufficient protein to support muscle repair and growth in response to muscle-damaging activities, and maintaining proper hydration to support performance and recovery [21,22]. Moreover, adopting comprehensive nutritional approaches, such as the Mediterranean diet, has been associated with improved physical performance and reduced inflammation and oxidative stress in athletes [6]. Therefore, emphasizing well-structured nutrition plans from an early stage may significantly benefit athletic development and performance in youth sports.

Based on the existing literature, a conceptual framework is proposed in which dietary patterns, BMI, and physical performance are interrelated during youth athletic development. Adherence to the Mediterranean diet may be associated with healthier dietary behaviours and lower BMI, which in turn are linked to physical performance outcomes such as sprinting and jumping ability. Considering this conceptual framework and the aforementioned gaps in literature, the main aim of this study was to explore associations between Mediterranean diet adherence score, BMI and selected physical performance measures in youth handball players, by identifying distinct player profiles through a clustering approach.

## 2. Materials and Methods

### 2.1. Design

Using a cross-sectional design, the study compared the physical performance of youth handball players during the competitive period between two clusters composed of players with similar Mediterranean diet adherence scores and BMI. Mediterranean diet adherence was evaluated using the 16-item KIDMED questionnaire and its total score. Also, the neuromuscular performance was assessed using the countermovement jump (CMJ) test, the 505 change of direction ability (505-CODA) test, linear straight sprints (i.e., 10, 20, 30 m), and isometric handgrip strength. All measurements were performed during a testing session in a handball court with the following conditions: 15–18 °C, 60–70% relative humidity. The standardized warm-up prior to the neuromuscular testing session consisted of running at a moderate intensity for 5 min, followed by 3 min of dynamic stretching, and ending with 7 min of jumps, changes of direction, and progressive runs. All participants had been familiarised with the questionnaires and physical assessments during an earlier phase of the competitive season. They were instructed to refrain from any strenuous physical activity for 48 h prior to data collection and were advised to arrive adequately hydrated for the testing session.

### 2.2. Participants

Thirty-five male youth Tier 2 [23] handball players (age: 15.6 ± 1.6 years, height: 170.9 ± 10.1 cm, body mass: 64.9 ± 15.0 kg; BMI: 22.1 ± 3.9 kg·m^−2^) belonged to the same handball academy and participated in the study. The inclusion criteria were to be part of the normal training sessions (three times per week of 60 min each) and official matches during the weekend and not having been injured during the month prior to data collection. Goalkeepers were excluded from the statistical analysis due to their special tactical role.

### 2.3. Mediterranean Diet Adherence (KIDMED Questionnaire)

Adherence to the Mediterranean diet was assessed using the KIDMED questionnaire [24]. This instrument consists of 16 items, including 12 questions positively associated with Mediterranean diet adherence (scored + 1) and four questions reflecting negative dietary behaviors (scored − 1). Items not endorsed by the participants were assigned a score of zero. The overall KIDMED score ranges from 0 to 12 points and is categorized into three levels of adherence: optimal (>8), intermediate (4–7), and very low (<3) [25].

### 2.4. Neuromuscular Performance

Countermovement jump (CMJ). Players completed two maximal-effort attempts, separated by a 2 min recovery period. Jump performance was assessed using a Kistler Quattro Jump force platform type 9290DD (Kistler Group, Winterthur, Switzerland), with data collected through MARS for Quattro Jump & KiJump 5.2 software (Kistler Group, Winterthur, Switzerland) at a sampling rate of 500 Hz. Athletes were instructed to keep their hands on their hips throughout the task, perform a maximal vertical jump, and land upright with knee flexion following ground contact [26]. This test was previously performed in youth handball players to assess the neuromuscular performance [27]. The jump height (cm) was recorded. The mean score was considered for statistical analysis, as it may be more sensitive than the best CMJ value for detecting fluctuations in neuromuscular function [28].

505-CODA test. Players were required to sprint to a turning line marked 15 m from the start line, pivoting 180° and sprinting back 5 m through the finish [29]. Test performance was defined as the elapsed time (s) required to sprint 10 m from the starting position, execute a 180° change of direction at the turning line, and cross the timing gate again. Timing was captured using a photocell system (Microgate Witty System, Microgate^®^, Bolzano, Italy) positioned 0.8 m above the ground at the start/finish line. Two attempts were completed for both left- and right-side turns, with a 90 s passive recovery between trials. The fastest recorded time (s) were retained for subsequent analysis.

Linear straight sprints. Participants completed two all-out 30 m linear sprint efforts (SPR30), separated by a 90 s passive recovery. Intermediate split times were obtained at 10 m (SPR10) and 20 m (SPR20). Sprints were initiated from a standing start, with players positioned 0.5 m behind the initial timing gate (Microgate Witty System, Microgate^®^, Bolzano, Italy), which was set at a height of 0.8 m. Sprint timing commenced automatically when the athlete crossed the first gate (0 m), and participants were instructed to sprint at maximal speed until crossing the finish line at 30 m [30]. For each sprint distance, the best performance was retained for statistical analysis. Sprint data were subsequently extracted using the Witty Manager v.1.5.45 software (Microgate^®^, Bolzano, Italy).

Handgrip strength. The handgrip strength was measured with a hand-held dynamometer (Camry Digital Hand Dynamometer, Camry Scale, South El Monte, CA, USA) as previously used in handball players [31]. This test is reliable and valid for assessing the isometric hand grip strength in the healthy population [32]. Participants applied a progressive and sustained force for a minimum of two seconds while performing the test with their dominant hand, with the elbow fully extended (180°). Two trials were completed, separated by a 90 s rest period, and peak force values were recorded in kilograms. The average of the two attempts was subsequently used for statistical analysis.

### 2.5. Statistical Analyses

Due to the presence of non-parametric data and the algorithm’s superior accuracy and reduced propensity for overfitting compared to conventional classification and regression tree modelling [33,34,35], a Random Forest analysis was employed for classification. Concretely, Random Forest clustering was used to automatically assign each player to a group based on proximity measures, allowing for the creation of similar groups and contrast between them. The clustering was conducted using the Random Forest algorithm in an unsupervised way, with the outcome variable set to null, and generated a proximity matrix which gives an estimate of the distance between observations based on the frequency of observations ending up in the same leaf node. To ensure the robustness of the unsupervised classification, the training parameters were configured by setting the number of trees to 1000 and cluster determination was fixed to k = 2, using the KIDMED index and BMI as input variables in the Random Forest clustering algorithm. This analysis resulted in two groups of players (Cluster 1 and Cluster 2) classified by similarity in their characteristics. The statistical model was optimized using the R-squared (R^2^) statistic and the Bayesian Information Criterion (BIC). The R^2^ statistic and the BIC value indicate whether the model is a good fit, so optimal clustering was established when maximizing the R^2^ statistic [36] and minimizing the BIC value [37].

The results are presented as frequencies, percentages (%), means, and standard deviations (SDs). To assess normality and equality of variances, the Kolmogorov–Smirnov statistic and the Levene test were used, respectively. The t-test for independent samples or the Mann–Whitney U-test was used to determine the differences in KIDMED index, BMI and neuromuscular performance between Cluster 1 and Cluster 2. Practical significance was assessed by calculating Cohen’s effect size (ES). ES values above 0.8, between 0.8 and 0.5, between 0.5 and 0.2, and below 0.2 were considered large, moderate, small, and trivial, respectively [38]. Data analysis was carried out using the JASP 0.13.1 software (University of Amsterdam, Amsterdam, The Netherlands). Statistical significance was established at *p* < 0.05.

## 3. Results

**Figure 1:**
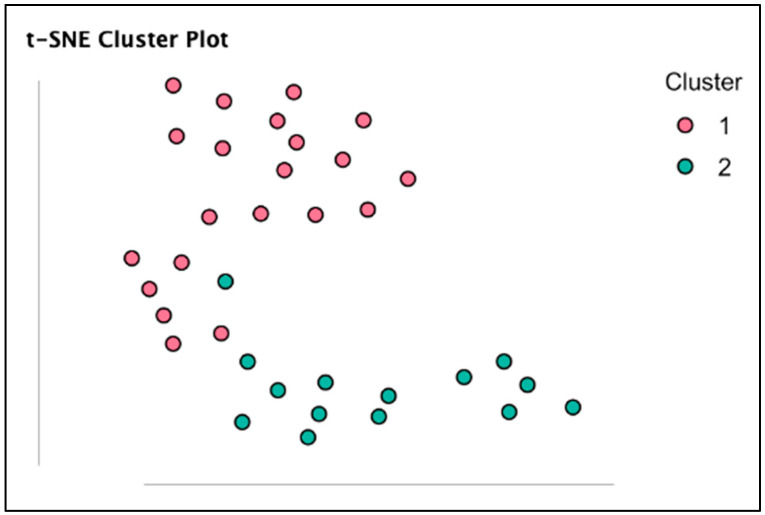
Depiction from the t-distributed stochastic neighbour embedding (t-SNE) cluster plot. The t-SNE two-dimensional space makes the axes uninterpretable. The t-SNE plot aims to convey the relative distances between players and clusters.

**Figure 2:**
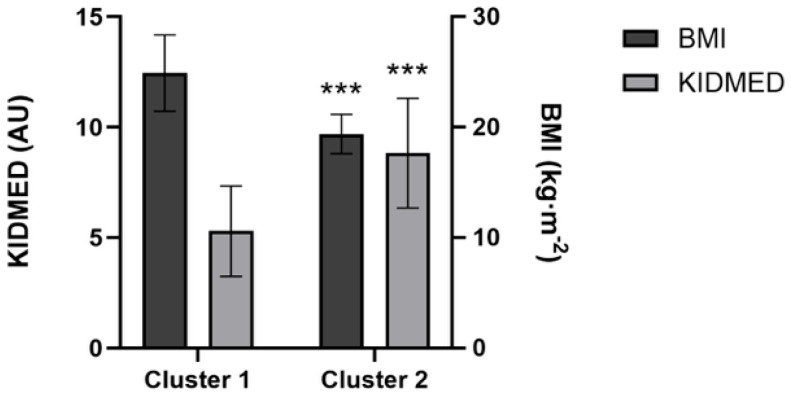
Cluster profile plot. *** Represent significant differences (*p* < 0.001) between clusters.

The variance explained by Random Forest clustering (R^2^) was 45.5% with a BIC value of 51.290. Seventeen players were assigned to Cluster 1 (higher BMI and worse KIDMED score), and eighteen players were assigned to Cluster 2 (lower BMI and better KIDMED score). Clusters are graphically presented in Figure 1. In addition, Figure 2 represents the significant differences between Clusters.

The results for each item of the KIDMED questionnaire for Clusters 1 and 2 are presented in Table 1. The total KIDMED score was 7.11 ± 2.88 AU, maximum = 13 AU and minimum = 1 AU.

**Figure 3:**
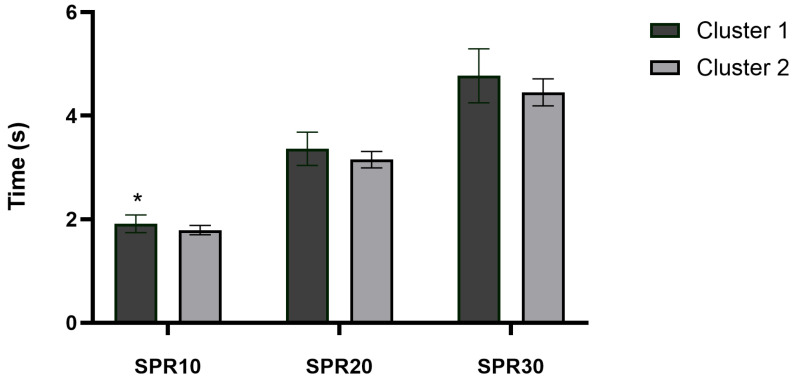
Sprint performance differences between clusters for the 10 (SPR10), 20 (SPR20) and 30 (SPR30) m sprint test. * Represent significant differences (*p* < 0.05) between clusters.

**Figure 4:**
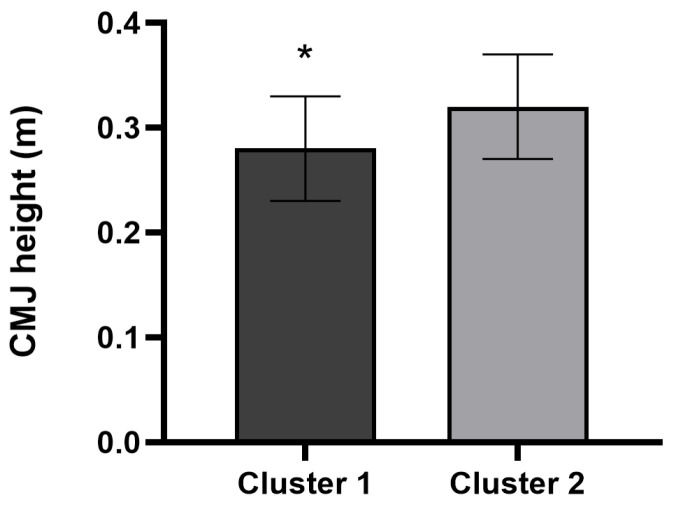
Mean jump height differences between clusters for the countermovement jump (CMJ). * Represent significant differences (*p* < 0.05) between clusters.

Cluster 2 players showed better SPR10 (*p* = 0.019; ES = 0.47; %Change = −6.44; Figure 3) and CMJ performance (*p* = 0.042; ES = 0.40, small; %Change = 12.77; Figure 4) in comparison to Cluster 1. However, between-cluster differences in SPR20 (*p* = 0.053; ES = 0.39; %Change = −6.34) and SPR30 (*p* = 0.051; ES = 0.39; %Change = −6.87) performance did not reach the conventional level of statistical significance, although both comparisons showed values close to the significance threshold. In addition, no significant differences were found in 505-CODA test (Cluster 1: 2.54 ± 0.24 vs. Cluster 2: 2.39 ± 0.15 s; *p* = 0.105; ES = 0.32; %Change = −5.87), and in handgrip strength (Cluster 1: 41.95 ± 9.93 vs. Cluster 2: 40.92 ± 8.04 kg; *p* = 0.869; ES = 0.04; %Change = −2.44).

## 4. Discussion

The main objective of this study was to explore associations between Mediterranean diet adherence score, BMI and selected physical performance measures in youth handball players, by identifying distinct player profiles through a clustering approach. We found that players in this study who adhered more closely to the Mediterranean diet and had lower BMI demonstrated healthier eating patterns and better sprint and jump performance. Nevertheless, the cross-sectional design of the study does not allow for causal relationships between these variables. So, these results highlight associations that may inform future research examining the role of healthy eating patterns, such as the Mediterranean diet, in youth sport players. 

Participants obtained a total score of 7.11 ± 2.88 AU in the KIDMED questionnaire, which is considered intermediate and indicates a need to improve the diet [25]. Previous studies involving adolescents, regardless of their participation in different sport modalities, have reported similar results. For example, a previous study involving youths aged 15–24 years residing in Spain found that 50.1% had intermediate values [25]. Likewise, another study reported a mean score of 7.6 ± 2.3 AU among 662 Spanish students aged 9–17 years [39]. In addition, other research carried out in sport contexts showed scores of 6.8 ± 2.3 AU in 16–17-year-old beach handball players [40] and scores of 5.7 ± 2.1 AU in 13–19-year-old swimmers [41]. Overall, it would be advisable to incorporate nutrition education interventions into daily routines to improve eating habits, nutrition knowledge, body composition, and physical performance in team-sport athletes [42]. On the other hand, comparing clusters for each KIDMED indicator, it was found that Cluster 1 goes to a fast-food restaurant more than once a week, skips breakfast more often, and consumes sweets and candy more often. This Cluster is characterized by a higher BMI and a lower KIDMED score. Therefore, it appears that these unhealthy dietary habits are associated with higher BMI, which is closely associated with poorer health status [9,11]. However, it is important to note that BMI is a crude measure that does not distinguish between fat mass and lean mass. In handball, particularly for pivots and backcourt players, a higher BMI may reflect greater muscle mass rather than excess adiposity. Thus, interpreting BMI solely as a negative nutritional outcome can be misleading. Future studies should incorporate direct body composition assessments (e.g., skinfolds, bioimpedance, DXA) and consider playing position to better contextualize these findings.

Physical condition is a fundamental component for maximizing performance in handball, a sport characterized by frequent high-intensity actions such as accelerations, jumps, changes of direction, pushing, and tackles, combined with handball-specific movements of passing, catching, throwing, checking, and blocking, with a great amount of body contact between players [20]. Cluster 2, composed of players with better dietary habits and lower BMI, demonstrated significantly superior performance in SPR10 (*p* = 0.019; ES = 0.47) and CMJ (*p* = 0.042; ES = 0.40), both of which are essential for explosive actions in handball gameplay. These results are consistent with previous findings that associate lower BMI with enhanced agility, speed, and vertical jump performance in athletes [43,44,45]. Nevertheless, the apparent “BMI–jump paradox” observed in Cluster 1 can be explained by mechanics: for a given absolute power, higher body mass reduces jump height unless power scales proportionally. Fat mass negatively affects sprint and jump performance, while lean mass tends to improve it—relationships that BMI cannot disentangle [46]. In this regard, it is important to remember that adherence to the Mediterranean diet has been associated with BMI because it reduces fat oxidation in previous literature [47]. Although causal effects cannot be inferred from the present study. Also, it is important to consider the potential influence of biological maturation when interpreting these findings. It is possible that maturation-related differences contributed, at least in part, to the observed cluster differences in BMI, SPR10, and CMJ performance. More biologically mature adolescents typically present greater fat-free mass, lower relative fat mass, and superior sprinting and jumping performance compared to their less mature peers.

Furthermore, in the context of handball, where quick accelerations and powerful jumps are essential for both offensive and defensive actions, players with these physical attributes may hold a competitive advantage [48,49]. However, no significant differences were observed between clusters in longer sprint distances (e.g., SPR20 and SPR30), CODA, or isometric handgrip strength. The lack of differences in longer sprints may be explained by the nature of handball gameplay, which rarely involves extended linear runs, while the CODA test may be more influenced by technical and coordinative factors rather than purely physical ones [29]. Regarding handgrip strength, its role in handball performance may be more closely related to specific actions such as physical contact and throwing, rather than to nutritional status or body composition. In fact, grip strength has been identified as a limiting factor in sports requiring manual manipulation and upper-body engagement, such as judo and handball [50]. Altogether, the use of cluster analysis based on both dietary and anthropometric variables provides valuable insights for coaching staff, enabling them to identify player profiles that combine healthier nutritional habits with physical attributes more conducive to high-level handball performance. As maturation status was not assessed, the extent to which the observed associations are attributable to dietary patterns, biological maturation, or their interaction cannot be determined. This reinforces the need for cautious interpretation of the results.

The results of the present study must be interpreted in light of the following limitations. First, the cross-sectional design does not allow us to establish causal inferences. Future experimental studies are needed to clarify the direct effects of Mediterranean diet interventions on BMI and physical performance in young athletes. Likewise, studies analysing chronic outcomes, as well as longitudinal follow-up research, would be advisable. Second, the relatively small sample size of male adolescent participants limits the generalizability of the findings. Specifically, although significant differences were identified in certain variables, the small-to-moderate effect sizes suggest that these findings should be interpreted with caution, as results in small groups can be sensitive to individual outliers. Thus, these results should be considered exploratory and require further validation in larger, more representative populations of different sex, age and team sports modalities. In addition, nutritional habits were assessed using a self-reported questionnaire, which relies on participants’ memory and honesty. Given that this study involved adolescent populations, this approach may be subject to recall bias and social desirability bias, potentially influencing the accuracy of the reported dietary behaviors. Additionally, although BMI provides a practical and economical method for categorizing body weight, it is only indirectly associated with body fat and does not account for lean mass. Therefore, future research should complement BMI with other assessments such as skinfold thickness, waist circumference, or bioimpedance to improve classification and interpretation, especially in sports where muscularity is advantageous. Finally, biological maturation was not measured, despite its well-established influence on both BMI and physical performance during adolescence. More advanced maturation is typically associated with greater muscle mass, enhanced neuromuscular performance, and improved sprint and jump ability. Consequently, maturation-related differences may have confounded the associations observed between dietary adherence, BMI, and physical performance. The absence of pubertal or maturation status assessment (e.g., maturity offset or peak height velocity) represents an important limitation, and future studies should prioritize the inclusion of maturation-related variables when examining performance outcomes in youth athletes.

## 5. Conclusions

The current results suggest that Random Forest clustering identified two distinct profiles among a cohort of youth male handball players based on Mediterranean diet adherence (KIDMED score) and BMI. Players characterized by higher KIDMED scores and lower BMI showed dietary patterns including lower fast-food consumption, less frequent breakfast skipping, and reduced intake of sweets and candy. In addition, this group exhibited better performance in SPR10 and CMJ. These results indicate associations between dietary adherence, BMI, and selected physical performance measures in this specific cohort. Given the cross-sectional design and the small sample size, these findings should be interpreted with caution and do not allow causal inferences. Nevertheless, the observed relationships may be useful for generating hypotheses and informing future longitudinal or interventional studies examining the role of nutritional patterns and body composition in youth handball performance.

From an applied perspective, these findings suggest that coaches and technical staff may benefit from monitoring specific, easily observable dietary behaviors such as breakfast consumption, lower fast-food intake, and reduced consumption of sweets and candy alongside basic anthropometric indicators such as BMI in youth handball players. Although causal relationships cannot be established, the identification of player profiles characterized by higher diet quality and healthier body composition, which were also associated with better sprint and jump performance, may help coaches to better understand interindividual differences in physical performance.

## Figures and Tables

**Table 1:** Description of item responses from the KIDMED questionnaire between clusters.

Item	Cluster 1 (n = 17)		Cluster 2 (n = 18)		Chi^2^ (*p*-Value)
	Yes n(%)	No n(%)	Yes n(%)	No n(%)	
Item 1. Consumes a fruit every day?	15(88.2%)	2(11.8%)	15(83.3%)	3(16.7%)	0.172 (0.679)
Item 2: Has a second fruit every day?	11(64.7%)	6(35.3%)	15(83.3%)	3(16.7%)	1.588 (0.208)
Item 3: Has a fresh or cooked vegetables once a day?	17(100%)	0(0%)	17(94.4%)	1(5.6%)	0.972 (0.324)
Item 4: Has a fresh or cooked vegetables more than once a day?	12(70.6%)	5(29.4%)	14(77.8%)	4(22.2%)	0.237 (0.627)
Item 5: Consumes fish regularly?	13(76.5%)	4(23.5%)	15(83.3%)	3(16.7%)	0.257 (0.612)
Item 6: Goes >1 day/week to a fast-food restaurant?	15(88.2%)	2(11.8%)	10(55.6%)	8(44.4%)	4.575 **(0.032)**
Item 7: Consumes pulses more than once a week?	7(41.2%)	10(58.8%)	7(38.9%)	11(61.1%)	0.019 (0.890)
Item 8: Consumes pasta or rice almost every day (5 or more/week)?	12(70.6%)	5(29.4%)	15(83.3%)	3(16.7%)	0.805 (0.369)
Item 9: Has cereals or grains (bread, etc.) for breakfast?	3(17.6%)	14(82.4%)	6(33.3%)	12(66.7%)	1.126 (0.289)
Item 10: Consumes nuts regularly (at least 2–3/week)?	9(52.9%)	8(47.1%)	11(61.1%)	7(38.9%)	0.238 (0.625)
Item 11: Uses olive oil at home?	15(88.2%)	2(11.8%)	15(83.3%)	3(16.7%)	0.172 (0.679)
Item 12: Skips breakfast?	14(82.4%)	3(17.6%)	8(44.4%)	10(55.6%)	5.381 **(0.020)**
Item 13: Has a dairy product for breakfast (yogurt, milk, etc.)?	14(82.4%)	3(17.6%)	14(77.8%)	4(22.2%)	0.114 (0.735)
Item 14: Has commercially baked goods or pastries for breakfast?	13(76.5%)	4(23.5)	8(44.4%)	10(55.6%)	3.736 (0.053)
Item 15: Consumes two yogurts and/or some cheese (40 g) daily?	8(47.1%)	9(52.9%)	9(50%)	9(50%)	0.030 (0.862)
Item 16: Consumes sweets and candy several times every day?	15(88.2%)	2(11.8%)	7(38.9%)	11(61.1%)	9.119 **(0.003)**

Bold used for highlighting significant differences between clusters.

## Data Availability

The data files that support the findings of this study are available from the corresponding author (D.C.) upon reasonable request.

## Abbreviations

The following abbreviations are used in this manuscript:

BIC	Bayesian Information Criterion
BMI	Body mass index
CMJ	Countermovement jump
CODA	Change of direction ability
ES	Effect size
SD	Standard deviation
SPR10	Time to cover 10 m distance
SPR20	Time to cover 20 m distance
SPR30	Time to cover 30 m distance
